# Heavy metal carcinogenicity: a scoping review of observational & experimental evidence

**DOI:** 10.3389/fonc.2025.1569816

**Published:** 2025-08-11

**Authors:** Julian Bailey, Shelly McFarlane, Icolyn Amarakoon

**Affiliations:** ^1^ Caribbean Centre for Research in Bioscience, University of the West Indies, Kingston, Jamaica; ^2^ Caribbean Institute for Health Research, University of the West Indies, Kingston, Jamaica; ^3^ Department of Basic Medical Sciences, Biochemistry Section, University of the West Indies Mona, Kingston, Jamaica

**Keywords:** cadmium, arsenic, carcinogenesis, colorectal cancer, prostate cancer, cervical cancer, heavy metals, environmental toxins

## Abstract

Environmental heavy metal pollutants are highly toxic and are usually of human origin. Studies have suggested a link between cadmium and arsenic carcinogenesis and geographical location. This review was conducted to explore the methodologies that have been used to determine the risk of carcinogenesis as it relates to cadmium & arsenic exposure as well as geographical location. A search of pertinent literature published up to December 2024 was conducted using the databases, PubMed, and EBSCO. The following MeSH terms were used primarily to search the databases, “heavy metals,” “cadmium,” “arsenic,” “carcinogenesis,” “malignancy,” and “toxicity.” Articles were removed if they were not closely related to the review topic. As evidenced in this review, there has been several research done over the years exploring the heavy metal exposure and the risk for carcinogenesis. The methodologies used to determine this risk are quite uniformed across the various studies. However, there is a paucity of studies dealing with the potential influence of geographical location in relation to the risk of carcinogenesis. This gap in knowledge shows that more work needs to be done to improve on the current knowledge of arsenic and cadmium and carcinogenesis.

## 1 Introduction

Studies done by researchers have aided in having a better understanding of individual cancers which has led to the development of screening programs, prophylactic treatments, and finally, the development of more targeted therapy. This improved understanding of the disease and treatment has yielded better prognoses and fewer side effects (1). When discussing cervical, prostate, and colorectal (CPC) cancers, investigators have acknowledged that none of these three cancers is driven by a single dominant mechanism of carcinogenesis. Instead, most research indicate that their development is often the result of multiple risk factors interacting synergistically to create an environment conducive to carcinogenesis (2–6). This review will focus on studies that explore the relationship/association between heavy metals and cancer. Various studies have been conducted looking at heavy metals in relation to cancer, from studies that simply looked at the presence of heavy metals in the blood of cancer patients, to studies that have looked at the mechanisms by which heavy metals could be causing cancers. Heavy metal pollution in the environment includes water, air, and soil and has been found to be mainly of human origins, these include fossil fuel burning, vehicle exhaust, waste incineration, and industrial processes such as mining and agriculture (7, 8). In addition to human origins, environmental heavy metal pollution also has naturally occurring origins such as infiltration and volcanic eruptions (7, 9). The most environmentally hazardous heavy metals have been found to include arsenic (As), cadmium (Cd), chromium (Cr), lead (Pb), mercury (Hg), and zinc (Zn). This is based on their significance to public health and their toxicity (7, 10). Though there is great interest in all the heavy metals cited, this review will focus on cadmium and arsenic specifically. When listing environmental mutagens as risk factors of cancers such as colorectal cancer, the compounds that are normally implicated are carcinogenic compounds that cause gene mutations, these include polycyclic aromatic hydrocarbons (from burning of gas, wood, garbage, and tobacco), heterocyclic amines, nitrosamines, and aromatic amines (11). Studies on the relationship between heavy metals and colorectal cancer are limited, however, there have been a few that compared the levels of heavy metals in the blood of colorectal cancer patients to a control group (healthy individuals). The result from one study found significant differences in trace elements and heavy metals levels between healthy subjects and metastatic colon cancer patients (12). Studies have hypothesized a possible relationship between heavy metals and the pathogenesis of prostate cancer, these studies have suggested that some heavy metals such as cadmium have estrogenic or androgenic abilities. Since prostate cancer progression has been surmised to be androgen-dependent, scientists have suggested that this may be a possible mechanism in which heavy metals are risk factors of prostate cancer (13, 14). Contrastingly, some studies have found no association between heavy metals and prostate cancer risk. A study done to explore the possible relationship between urinary arsenic & blood cadmium, lead & mercury levels & prostate specific antigen (PSA) did not find any association between these heavy metal levels in the body and PSA (15). On the other hand, other studies found evidence that suggested a plausible relationship with metals such as zinc, cadmium, and arsenic levels in the body & the risk of prostate cancer (13, 16).

A study done in Jamaica in 2004 explored the levels of cadmium concentrations in autopsied kidneys (6.7 to 126 mg/kg^−1^, with a mean of 43.8 mg/kg^−1^) and livers (0.3 to 24.3 mg/kg^−1^, with a mean of 5.3 mg/kg^−1^) of deceased Jamaicans. This study found that the levels seen, especially in the kidney samples were much higher than values that were reported in other countries in the world (17–19). The study went on to specify that the values seen in Jamaica was second only to values seen in Japan (20). The cases sampled in this study were taken from areas in Jamaica where the concentration of cadmium in the soil was low compared to other areas of the country (21). It is of great interest to examine what the values would be when analyzed in individuals residing in areas such as central part of the country where the soil content of heavy metals has been documented to be above world averages (21, 22). A recent study done in Jamaica demonstrated that there were higher number of cervical, prostate and colorectal (CPC) cancer cases in areas in Jamaica with historically reported high levels of heavy metals such as cadmium (23). Additionally, one location of interest in Jamaica, is a rural parish in the southwestern section of the island, St. Elizabeth that has been found to have the highest concentrations of arsenic in the country, with one specific farming community being described as an arsenic anomaly due to the abnormally high levels of arsenic in the soil (21, 24).

In this review, we have considered the potential factors that may result in cadmium and arsenic exposure as well as the methods of metal induced carcinogenesis. While there have been a few studies that have explored the association between cadmium, arsenic and CPC cancers, the knowledge of the exact mechanism of action remains unclear. We seek to explore and understand the existing literature as it relates to heavy metal carcinogenicity and are especially focused on exposure as it relates to geographical location.

### 1.1 Review questions

The main concepts in the review questions and the search strategy were informed by the PCC (Population (or participants)/Concept/Context) framework (25). Considering the existing gap in the literature this review seeks to answer the following research questions:

1. What are the methodologies used to determine carcinogenesis risk of heavy metals in studies that explored the association of cervical, prostate, and colorectal cancer risk with cadmium and arsenic?
2. Does geographical location influence the relationship between cadmium and arsenic exposure and cervical, prostate, and colorectal cancer risk?

## 2 Materials and methods

This scoping review has been conducted in accordance with the JBI methodology for scoping reviews (26). The PRISMA-ScR checklist was used to inform the reporting of this review (27).

### 2.1 Search strategy

A search strategy was developed to search the PubMed and EBSCO host databases to retrieve articles on the cancer risk and cadmium & arsenic exposure. Articles retrieved were limited to those which had free full text available, human species studies, and English language for articles (**Table 1**).

**Table 1:** Inclusion and exclusion criteria used in search strategy.

Inclusion criteria	Exclusion criteria
1. Studies that investigate cadmium and arsenic as the heavy metal of interest 2. Studies that examine potential predictors, risk factors, or determinants of cervical, prostate or colorectal cancer in relation to cadmium and arsenic 3. Peer-reviewed articles 4. Studies conducted locally, regionally or internationally	1. Articles that were not in English 2. Studies focusing on cancers that were not cervical, prostate or colorectal cancer 3. Studies exploring heavy metals that did not include cadmium or arsenic 4. Studies where cancer and carcinogenesis was not the primary condition 5. Studies explored benign tumors only but did include malignant tumors. 6. Articles that focused on animal Studies 7. Systematic Review articles

There were no minimum or maximum date used in the search since the authors wanted to include as many studies as possible considering specificity of the study search. The primary keywords used in the search were “heavy metals,” or “arsenic” and “cadmium” with “carcinogenesis,” “cervical cancer,” “colorectal cancer,” and “prostate cancer.” The detailed search phrase can be found in Appendix 1 and 2.

The articles were selected to explore methodologies used to determine heavy metals exposure, their association with cervical, prostate and colorectal cancer risk, and subsequently, the potential impact of geographical location of the population. The inclusion and exclusion criteria are shown in **Table 1**.

**Figure 1:**
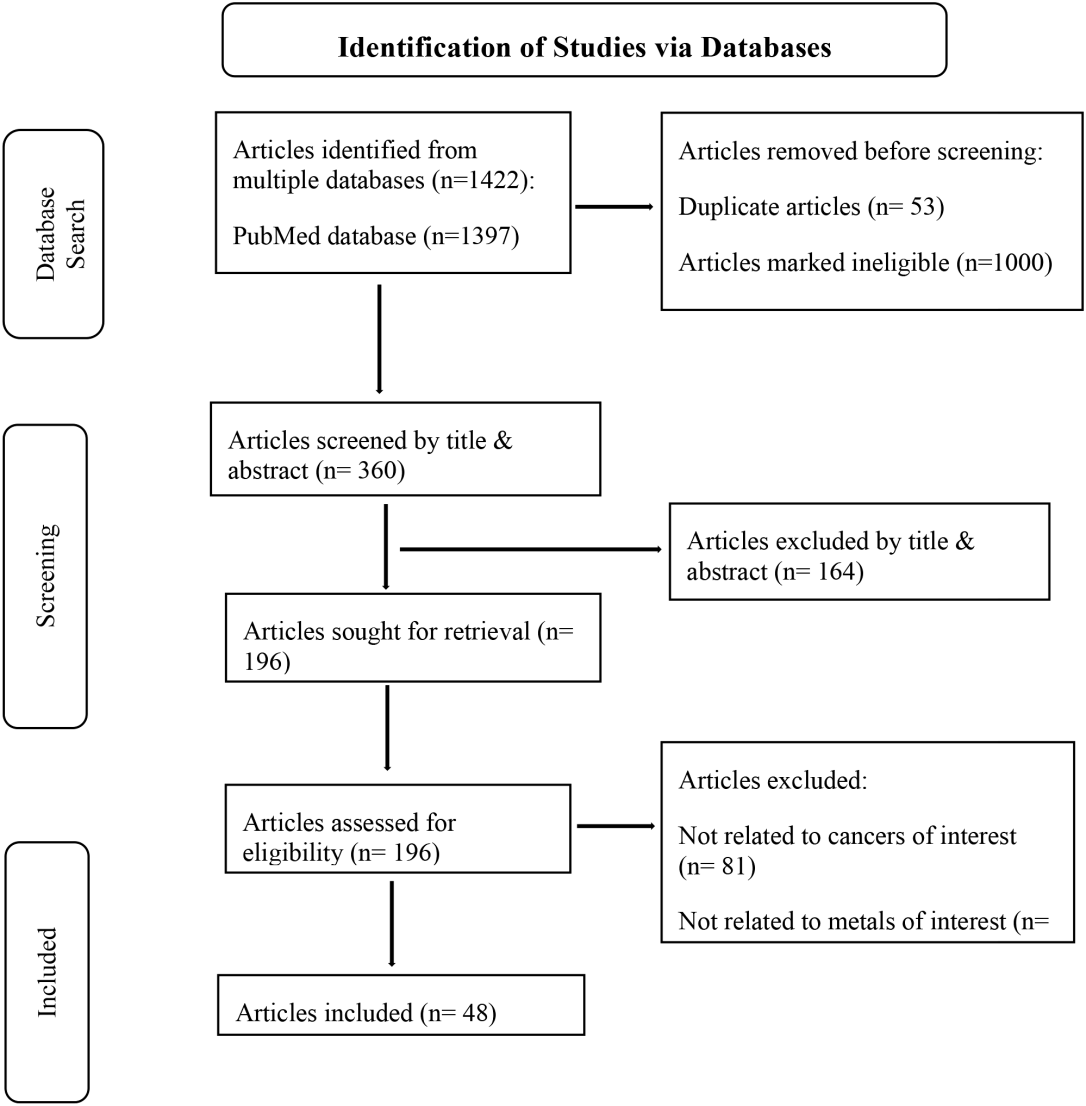
PRISMA-ScR diagram highlighting summary of the inclusion and exclusion process (27).

The search terms used are listed in Appendix 1 and yielded 1397 results from PubMed and 25 from EBSCO (total 1422 articles which contained 53 duplicates). After the duplicates were removed, 1000 articles were automatically marked ineligible, and 9 articles were removed for other reasons (such articles that were outdated based on information included in more recent publications). The titles and abstracts of the remaining 360 articles were then screened by a single author according to the predetermined inclusion and exclusion criteria outlined in **Table 1**. After the title and abstract screening, 196 articles were eligible for full text review and ultimately 48 articles were included in this study for analysis. The management of citations throughout this process was facilitated using Mendeley reference manager. To address the research questions, a thematic approach/strategy was used to provide a comparative description of the studies. A summary of the search and inclusion & exclusion process is presented in the Preferred Reporting Items for Systematic Reviews and Meta-analyses extension for scoping review (PRISMA-ScR) flow diagram (**Figure 1**) (27).

## 3 Results

Following the search strategy, the included articles were sorted into two categories by the reviewers. The first category was observational studies (**Table 2**) and the second category was experimental studies (**Table 3**). The articles had publication dates ranging from 2000 to 2024.

**Table 2:** Observational studies.

References	Population	Study design	Exposure	Outcome	Geographical location	Sample type	Metal	Main findings
(31)	66 patients & 64 healthy controls	Case Control	Environmental toxins	Prostate Cancer	Poland	Whole Blood	Fe, Ni, As, Cd, Pb, Hg, Cr, Zn	No statistically significant difference between place of residence, work environment, alcohol and nutrition. Patients with cancer had a significantly higher concentration of arsenic.
(28)	147 patients= 88 males & 59 females	Cross-Sectional	Environmental toxins	Colorectal Cancer	Pakistan	Tumor & non-tumor tissues	Ca, Na, Mg, K, Zn, Fe, Ag, Co, Pb, Sn, Ni, Cr, Sr, Mn, Li, Se, Cd, Cu, Hg, As.	Significantly higher levels of Zn, Pb, Cr & Cd in tumor tissues when compared to nontumor tissues. Significantly higher levels of Ca, Na, Mg, Fe, Sn & Se in non-tumor tissues when compared to tumor tissues.
(29)	56,633 colorectal cancer patients & 77,679 breast cancer patients	Correlational	Ambient concentrations of heavy metals	Colorectal & Breast Cancer	Kentucky, USA	N/A	As, Cd, Cr, Ni	Higher ambient metal exposures were associated with higher odds of residing in breast and colorectal cancer hotspots. Cd had the strongest association with residence in cancer hotspots
(30)	256 specimens from patients	Cross Sectional	Smoking Habits	Prostate Cancer	Kentucky, USA	Serum, urine, & tissues	As, Cd, Pb, Cr, & Ni	Significantly higher levels of As & Cd seen in malignant tissue when compared to the adjacent healthy tissues. Significantly high urinary levels of Cd seen in 70% of prostate cancer cohort when compared to the CDC-reported cutoff values Significantly high urinary levels of As seen in 2.3% of prostate cancer cohort when compared to the than the CDC-reported cutoff values
(32)	82 prostate cancer patients, 93 benign prostatic hyperplasia (BPH) patients 98 controls	Prospective Case Control	Environmental Toxins	Prostate Cancer & BPH Risk	Nigeria	Blood & Urine	Cd & Zn	Every 10-fold increase in urinary Cd & urinary Cd/Zn ratio was associated with increased risk of prostate cancer when compared to the cases of BPH Blood Cd levels were lower than the limit of detection in 27% of prostate cancer cases, 20% of BPH cases & 90% of the control group. No statistically significant association observed between urinary Cd, urinary Zn or blood Zn in prostate cancer cases when compared to the control group during the multivariate analysis.
(91)	230,540 Patients	Retrospective Cohort	Air cadmium	Prostate Cancer Aggressiveness	USA	N/A	Cd	Statistically significant associations between air Cd and aggressive prostate cancer in nonmetropolitan counties with an urban population of 20,000 to 250,000.
(46)	3,792 men and women (45–75 years of age) from 13 American Indian communities.	Prospective cohort study	Long-term Cd exposure in individuals followed for up to 20 years	All cancer	Arizona, Oklahoma, and North and South Dakota, USA	Urine	Cd	No significant association found between Cd and mortality from multiple cancers including prostate and colorectal cancer. Significant association between Cd exposure and mortality from lung and pancreatic cancers even after adjusting for smoking status & cigarette pack-years at baseline
(103)	22 African American & 30 European American men	Cross Sectional	Environmental Toxins	Prostate Cancer	Michigan, USA	Prostate cancer tissue	Cd	African American men had a significant positive correlation between tumor tissue cadmium content and androgen receptor expression European Americans showed a non-significant negative correlation between the two
(33)	18 patients & 25 control	Case Control	Environmental Toxins	Prostate Cancer	Not specified	Tissue	Cd	No statistically significant difference in Cd concentration between the cases and controls.
(34)	61 patients & 61 healthy controls	Case Control	Environmental Toxins	Prostate Cancer	Michigan, USA	Peripheral Blood	As, Cd, Cu, Pb, or Zn	No significant differences in mean metal levels or CT-like activity between cases and controls
(35)	261 patients & 267 controls	Case Control	Environmental Toxins	Prostate Cancer	Taiwan	Blood & Urine	Cd	No statistically significant difference in blood Cd levels between cases and controls
(38)	40 patients with PSA recurrence and 40 patients without recurrence	Case Control	Environmental Toxins	Prostate Cancer Recurrence	USA	Tissue	Cd, Fe, Zn, Se	lower concentrations of Fe & Zn were associated with PSA recurrence. No statistically significant differences in Cd & Se levels seen in recurrence cases, when compared to nonrecurrences
(36)	115 patients & 227 age-matched controls	Case-Control	Environmental Toxins	Prostate Cancer Risk	Maryland, USA	Toenail	Cd & Zn	Prostate cancer risk did not increase with increasing concentrations of cadmium and did not decrease with increasing concentrations of zinc.
(41)	Communities served by water systems	Ecological Study	Environmental Toxins	County level prostate cancer incidence	Illinois, USA	Drinking water from community water systems	As	Counties with higher mean arsenic levels in community water systems had significantly higher prostate cancer incidence
(47)	139 patients & 400 healthy controls	Cohort Study	Environmental Toxins	Arsenic Speciation in prostate cancer	Canada	Toenail & Urine	As	Mean percent monomethylarsonic acid (%MMA), a key intermediate in arsenic metabolism was significantly lower in toenails from prostate cancer cases compared to controls. Secondary methylation index (SMI), a measure of the body’s efficiency in methylating arsenic, was significantly higher in toenails from prostate cancer cases compared to controls.
(92)	78,914 patients	Cross-Sectional	Heavy metals in the air	Prostate Cancer survival rate	Pennsylvania, USA	N/A	Cd & As	Increasing average & cumulative air concentrations of As & Cd were statistically significantly associated with lower overall and prostate cancer-specific survival among prostate cancer cases
(13)	141 patients & 114 healthy controls	Case-Control	Environmental Toxins	Prostate Cancer Risk	Singapore	Serum	Mn, Cu, Zn, As, Se, Sb, Co, Cu, Cd, Pb	Significant & positive association of As, Zn, Mn, Sb with prostate cancer risk.
(42)	Cervical specimens	Ecological Study	As in well water	Cervical Cancer	Bangladesh	Tissue	As	No significant increase in cervical cancer seen with increasing As concentration. Strong dose response seen for poorly differentiated cancer with increasing As exposure.
(37)	51 & 48 healthy controls	Case-Control	Environmental Toxins	Hematological Derangement in Cervical Cancer	India	Blood	As	Malondialdehyde, a critical marker of oxidative stress in arsenic induced hematological damage, was increased in high arsenic concentration when compared to patients with low or no arsenic concentration. Red Blood Cell count was lower in patients with high blood arsenic levels compared to those without arsenic. Hemoglobin levels were lower in patients with high blood arsenic levels compared to those without arsenic.
(104)	1,332,237 (female) and 1, 227, 516 (male) across 18 cities	Not stated	Environmental Toxins	Incidence and morbidity rate of colorectal cancer	Silesia Province, Poland	N/A	Cd	The highest concentrations of Cd were recorded in Piekary Slaskie, Zabrze and Bytom The lowest concentrations of Cd were recorded in Bielsko- Biala and Jastrzebie Zdroj. The highest incidence of colorectal cancer was found in women in Czestochowa, Piekary Slaskie and Sosnowiec, & the lowest in Myslowice, Zory and Jaworzno
(105)	25 patients (16 men & 9 women)	Cross-Sectional	Environmental Toxins	Large intestine cancer (Adenocarcinoma mucinosum)	Poland	Tissue	Cd, Zn, Cu, Sn, Ca, Mg, & Fe	Higher levels of Cu, Se Mg seen in malignant tissue compared to normal tissues. No significant difference seen in the levels of Zn, Ca, Cd & Fe in malignant tissue when compared to non-malignant tissue.
(43)	1184 colon cancer cases (656 males & 528 females)	Ecological Study	Heavy metals in rice milled in factories of interest	Incidence rate of colorectal cancer	Golestan Province, Iran	Rice	Cd, Ni, Co, Cu, Sn, Pb & Zn	No significant association reported for Cd concentrations. Inverse association between high selenium concentrations and colon cancer incidence in men. Positive association between cobalt and colon cancer
(44)	Patients served by community water system in 19 counties	Ecological Study	Arsenic in drinking water	Incidence, prevalence, & mortality rates of colorectal, bladder & kidney cancers in population served by community water systems	USA	Drinking water from community water systems	As	When weighting by population served, there were stronger, positive associations between bladder, colorectal, and kidney cancers & aggregated cumulative county-level arsenic concentration over the 11-year time period, compared to the unweighted exposure assessment method.
(45)	N/A	Ecological Study	Arsenic in drinking (well) water	Colon Cancer	Taiwan	N/A	As	Gradual decline in mortality for colon cancer in males, but not in females after the improvement of the drinking-water supply system (elimination of arsenic from artesian well water).
(12)	40 patients & 29 healthy controls	Case-Control	Environmental Toxins	Colon Cancer	Turkey	Serum	Cu, Mg, Pb, Cr, Zn, Se, Mn, &Cd	Higher levels of Cu, Mg, Pb, Cr, Zn, Mn and Cd were seen in colon cancer patients when compared to healthy controls
(48)	41,089 patients	Prospective Cohort	Dietary Cd	Prostate Cancer	Sweden	N/A	Cd	Positive association between dietary cadmium exposure & overall prostate cancer cases

**Table 3:** Experimental research studies.

References	Methods	Exposure	Outcome	Metal	Main Findings
(49)	Cell Assay	Chronic cadmium exposure of non-malignant RWPE-1 & PWR1E cells and PCa cells (DU 145)	Cell proliferation Apoptosis Modulation of Erk/MAPK pathway genes	Cd	Survival, proliferation and colony formation with inhibition of apoptosis in RWPE1 & PWR1E cells. Significantly increased proliferation and decreased apoptosis in DU 145 cells.
(60)	Cell Assay	Acute & chronic cadmium exposure in non-malignant RWPE-1 (prostate epithelial) cells	Activation of NOX1 complex ROS generation Endoplasmic reticulum stress	Cd	Facilitation of NOX1 assembly by activating its cytosolic regulators p47phox and p67phox.
(53)	Cell Assay *in vitro*	Cd-exposed colorectal cancer cells (HT116 and SW480) treated with resveratrol	Reversal of cadmium-promoted migration, invasion, and expression of epithelial-mesenchymal transition (EMT) related markers	Cd	Cd promoted the migration and invasion of colorectal cancer cells. Cd up-regulated the expressions of N-cadherin, vimentin, and ZEB1, while it down-regulated expression E-cadherin. The migratory and invasive ability of Cd-exposed colorectal cancer cells decreased as the concentration of resveratrol increased. High concentrations of resveratrol remarkably decreased the expressions of ZEB1, vimentin, and N-cadherin & increased the expressions of E-cadherin in the Cd-exposed cells.
(51)	Cell Assay *in vitro* analysis	Chronic cadmium exposure in normal prostate epithelial (PWR1E and RWPE1) cells	Malignant transformation of normal prostate epithelial cells	Cd	Induction of tumorigenic attributes (increased wound healing, migration and invasion capabilities) in both cell lines. Induction of oncogenes (P110α, Akt, mTOR, NFKB1 and RAF). Attenuation of tumor suppressor (TS) genes in Cd-exposed RWPE1 cells. Enrichment of prostate cancer related pathways cells exposed to Cd.
(106)	Cell Assay	Chronic cadmium in human BPH cells	Transformation of human BPH cells into prostate cancer	Cd	Cd induced substantial growth changes & morphology of BPH cells
(62)	Cell Assay	Cadmium in human (colorectal) adenocarcinoma cells (HT-29)	Increased migratory capacity of human colorectal cancer cells	Cd	Time-dependent increase in cyclooxygenase-2 (COX-2) expression in cells exposed to Cd. Time dependent significant promotion of wound healing activity in cells exposed to Cd. Significant attenuation of Cd-induced migration following pre-treatment of HT-29 cells N-Acetylcysteine (a ROS scavenger).
(56)	Cell Assay	Cadmium in Cadmium-transformed prostate epithelial (CTPE) cells & normal prostate epithelial (RWPE-1) cells	Genetic signatures of CTPE cells. The potential molecular signaling involved in their malignant transformation	Cd	Genes which are mainly involved in cell proliferation and focal adhesion (SATB1 and EYA2) expression were significantly upregulated in CTPE cells when compared to RWPE-1 cells. Genes involved apoptotic stimuli and disrupting the cell cycle of the cells (PITX2, PDLIM4, FABP5) were significantly downregulated in CTPE cells compared to RWPE-1 cells
(61)	Cell Assay	Chronic cadmium in normal prostate epithelial cells (RWPE-1)	Defective autophagy due to induction of endoplasmic reticulum.	Cd	Significant induction of ER-stress positive cells was seen in Cadmium-transformed prostate epithelial (CTPE) as compared to healthy RWPE-1 cells. Upregulation of autophagy-related 5 (Atg5) expression with acute exposure of Cd. Downregulation of Atg5 expression in chronically exposed RWPE-1 cells (2, 5, 8 Months) and CTPE cells.
(73)	Cell Assay	Cadmium in normal prostate epithelial cells (RWPE-1) & Cadmium-transformed prostate epithelial (CTPE) cells	Pro-survival function promotion of autophagy by induction of Plac8	Cd	Significant increase in cell death was observed in Cd-exposed RWPE-1 cells, compared with CTPE cells.
(81)	Cell Assay	Psoralidin in Cadmium-transformed prostate epithelial (CTPE) cells and normal prostate epithelial cells (RWPE-1)	Prevention of Cd-induced prostate carcinogenesis by inhibition of autophagy using Psoralidin	Cd	Significant inhibition of CTPE cell growth and proliferation by Psoralidin. No significant inhibition of cell growth seen in normal prostate epithelial cell. Inhibition of Placenta Specific 8 expression which resulted in growth inhibition.
(52)	Cell Assay	KRAS activation in Cadmium-transformed prostate epithelial (CTPE) cells	Development & maintenance of Cd-induced malignant transformation of CTPE cells	Cd	KRAS knockdown (KD) reduced stimulated RAS/ERK and PI3K/AKT signaling pathways & significantly mitigated multiple physical and molecular malignant cell characteristics. No reversal of miRNA expression (originally downregulated by Cd transformation) was seen with KRAS KD.
(54)	Cell Assay	Cadmium in normal prostate epithelial cells (RWPE-1)	Modulation of oncogene and tumor suppressor gene expression in human prostate cells	Cd	Rapid increase in c-myc & p53 mRNA levels with initial Cd exposure followed by a slight decline with continued exposure. Steady increase in c-jun mRNA levels as the time of Cd-exposure increased.
(55)	Cell Assay	Cadmium in 1LN prostate (cancer) cells.	1LN prostate cells proliferation & and division	Cd	Increased [^3^H] thymidine uptake and cell number following Cd-exposure. Increased transcription factors (NFkB and CREB). Increased expression proto-oncogenes involved in cell growth and regulation (c-fos & c-myc)
(50)	Cell Assay	Arsenic–transformed prostate epithelial (CAsE-PE) compared to normal prostate epithelial (RWPE-1) cells (control)	As-induced prostate cancer progression due to chronic activation of Ras/MAPK pathway signaling	As	Increased expression of phosphorylated MEK1/2 and Elk1 in CAsE-PE cells. Significant increase in K-Ras protein level in CAsE-PE cells when compared to RWPE-1 cells. HER-2/neu protein in CAsE-PE cells was expressed at significantly greater levels when compared to RWPE-1 cells (control).
(107)	Cell Assay	Arsenic–transformed prostate epithelial (CAsE-PE) compared to normal prostate epithelial (RWPE-1) cells (control)	Molecular events occurring during arsenic-induced malignant transformation	As	Significant reduction in genomic DNA methylation was seen in CAsE-PE cells. Over expression of the K-ras gene seen in CAsE-PE cells. Significant increase in Matrix metalloproteinase-9 (MMP-9) activity in arsenic-exposed cells compared to control.
(57)	Cell Assay	Arsenic–transformed prostate epithelial (CAsE-PE) compared to normal prostate epithelial (RWPE-1) cells (control)	Malignant transformation and acquisition of androgen Independence by normal cells	As	Significant increase in proliferation seen in CAsE-PE cells in when compared to control cells. CAsE-PE cells a more rapid growth rate in steroid-depleted medium when compared to control cells grown in the same condition.
(58)	Cell Assay *in vivo* & *in vitro*	Androgen, cadmium, and arsenic in prostate cells (PZ-HPV-7, CA-HPV-10, LNCaP, PC-3, and DU145)	Expression of metallothioneins (MT1, MT2, & MT3) & their resulting effects on cell proliferation, invasion, and tumorigenesis	As & Cd	Androgen, cadmium, and arsenic enhanced gene expression of MT1/2 & MT3 in prostate carcinoma poorly metastatic prostate carcinoma cells (LNCaP). Significant increase in cell proliferation, invasion, and tumorigenic in highly invasive prostate cancer cells (PC-3) with overexpression of MT3
(59)	Cell Assay	Chronic cadmium in prostate cancer cells (PC-3 & DU145)	Epithelial-mesenchymal transition (EMT) & malignant phenotypic changes of cells.	Cd	Dose-dependent significant increase in migration in PC-3 cells (highly metastatic) & DU145 cells (moderately metastatic) chronic Cd-exposure. Dose-dependent significant downregulation of epithelial marker (E-cadherin) in chronic Cd-exposed cells. Dose-dependent significant upregulation of vimentin, a mesothelial marker in chronic Cd-exposed cells.
(88)	Cell Assay	Arsenic trioxide in cervical cancer cells (SiHa)	Anti-cancer changes in cervical cancer cells	As	Inhibition of cell proliferation and invasion of cervical cancer cells. Time and dose dependent ROS production. Promotion of cervical cancer cell apoptosis via HIF-1α.
(86)	Cell Assay	Arsenic trioxide in cervical cancer cells (HeLa, SiHa, CaSki & C33A)	Anti-cancer changes in cervical cancer cells	As	Dose dependent inhibited cervical cancer cell proliferation. Greater As-induced growth inhibition seen in HPV-negative C33A cells when compared to that seen in HPV-positive cervical cancer cells.
(85)	Cell Assay	Arsenic trioxide in cervical cancer cells	Arsenic trioxide delivered using liposomal nanotechnology in HPV positive cervical cancer	As	Arsenic trioxide delivered using liposomal technology was more effective in reducing protein levels of HPV-E6 and inducing cell apoptosis when compared free arsenic trioxide
(87)	Cell Assay	Arsenic trioxide in cervical cancer (HeLa, SiHa, Caski) cells	Reduction in the invasive & metastatic properties of cervical cancer cells	As	Inhibition of tumor cells attachment to Fibronectin and Matrigel Reduction in cell motility & inhibition of tumor invasion potential.

### 3.1 Observational studies

**Figure 2:**
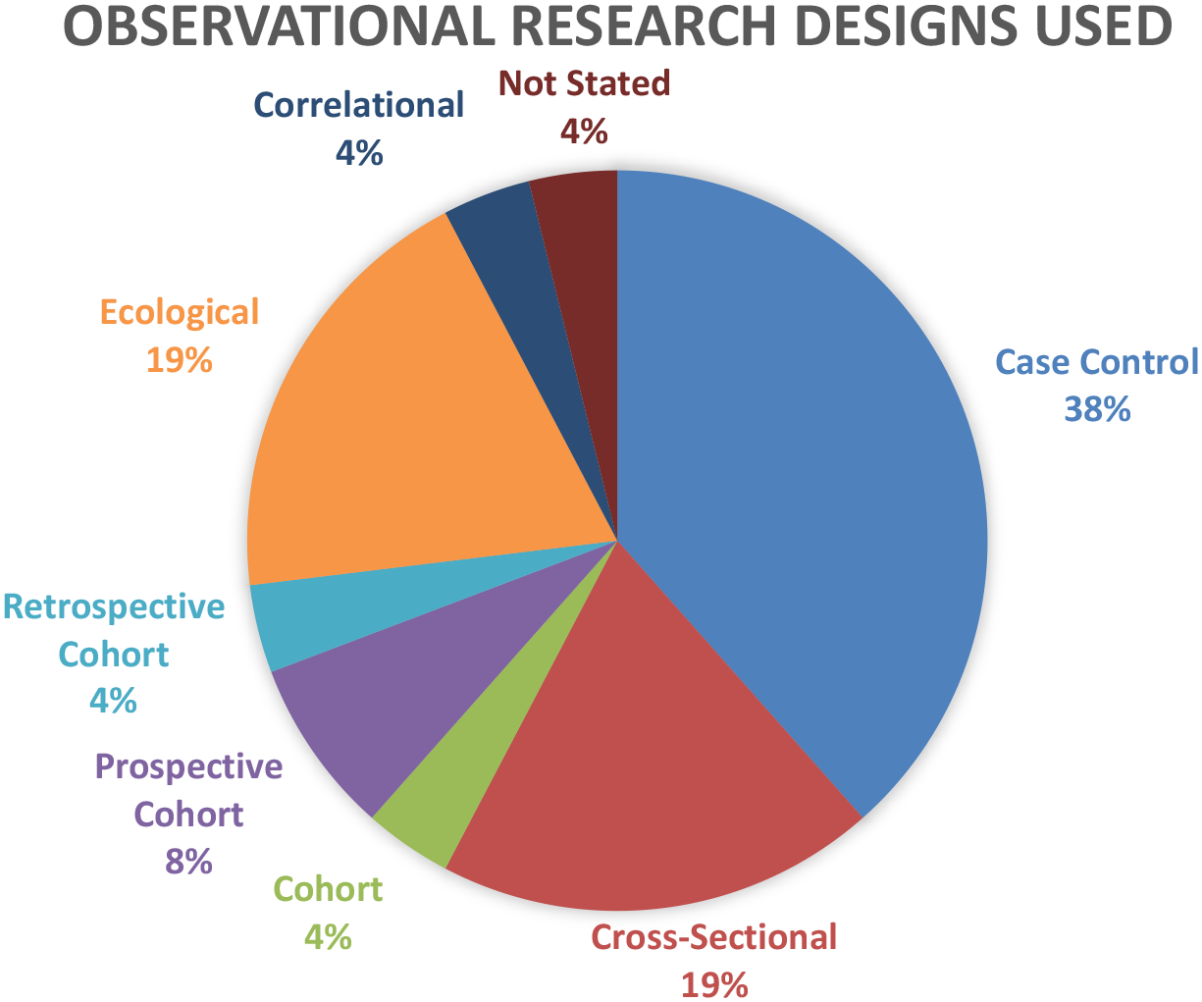
Case control studies accounted for 38% of the total observational studies used by researchers, while ecological and cross-sectional studies both accounted for 19% each.

In total there were 26 observational studies (**Table 2**), comprising approximately 3 million individuals from 12 countries identified and included in this review. There were various study designs used including cross sectional, case-control, cohort and ecological (**Figure 2**). In studies where cancer tissue was compared to non-cancer tissue in the same patient, there were significantly elevated levels of heavy metals in the cancer tissues (28–30). Case control studies which compared the concentration/levels of cadmium or arsenic in the biological samples of healthy controls and patients were ambivalent (12, 13, 31–37). While some studies showed significant differences between the concentration of the metals in the control group and patients (12, 13, 31), other studies reported no statistically significant difference (33–36). One case control study explored the risk of prostate cancer recurrence by comparing the levels of various metals in resected tissue samples of patients with PSA recurrence and those without recurrence (38). The study found no statistically significant differences when the levels were compared. Another study compared the level of arsenic, estimated malondialdehyde (MDA), hemoglobin, and red blood cell count in patients with cervical cancer and healthy controls to explore if there is a correlation between arsenic and MDA, hemoglobin and red blood cell count (37). The results of the study found that MDA was increased in high arsenic concentration when compared to patients with low or no arsenic concentration. Additionally, the red blood cell counts and hemoglobin levels of patients with high blood arsenic levels was found to be lower than patients without arsenic. Malondialdehyde is a reactive compound that is a physiological metabolite of lipid peroxidation of polyunsaturated fatty acids and is a critical marker of oxidative stress in arsenic induced hematological damage (37, 39, 40). A few ecological studies explored the potential relationship between the metals of interest in various environments and prevalence, incidence, & mortality of CPC cancers (41–45). One study that explored the arsenic levels in drinking water found that areas with higher mean arsenic levels in community water systems had significantly higher prostate cancer incidence (41). Another study that explored the possible relationship between the arsenic content in well water used for drinking and cervical cancer found a strong dose response seen for poorly differentiated cancer with increasing arsenic exposure, however, it did not find any significant increase in cervical cancer cases when the concentration of arsenic was increased (42). There were 4 cohort studies (46–48), 3 being prospective and one retrospective. The cohort studies made suggestions on the relationship between the metals of interest and the mortality, aggressiveness, incidence of the CPC cancers.

### 3.2 Experimental studies

**Figure 3:**
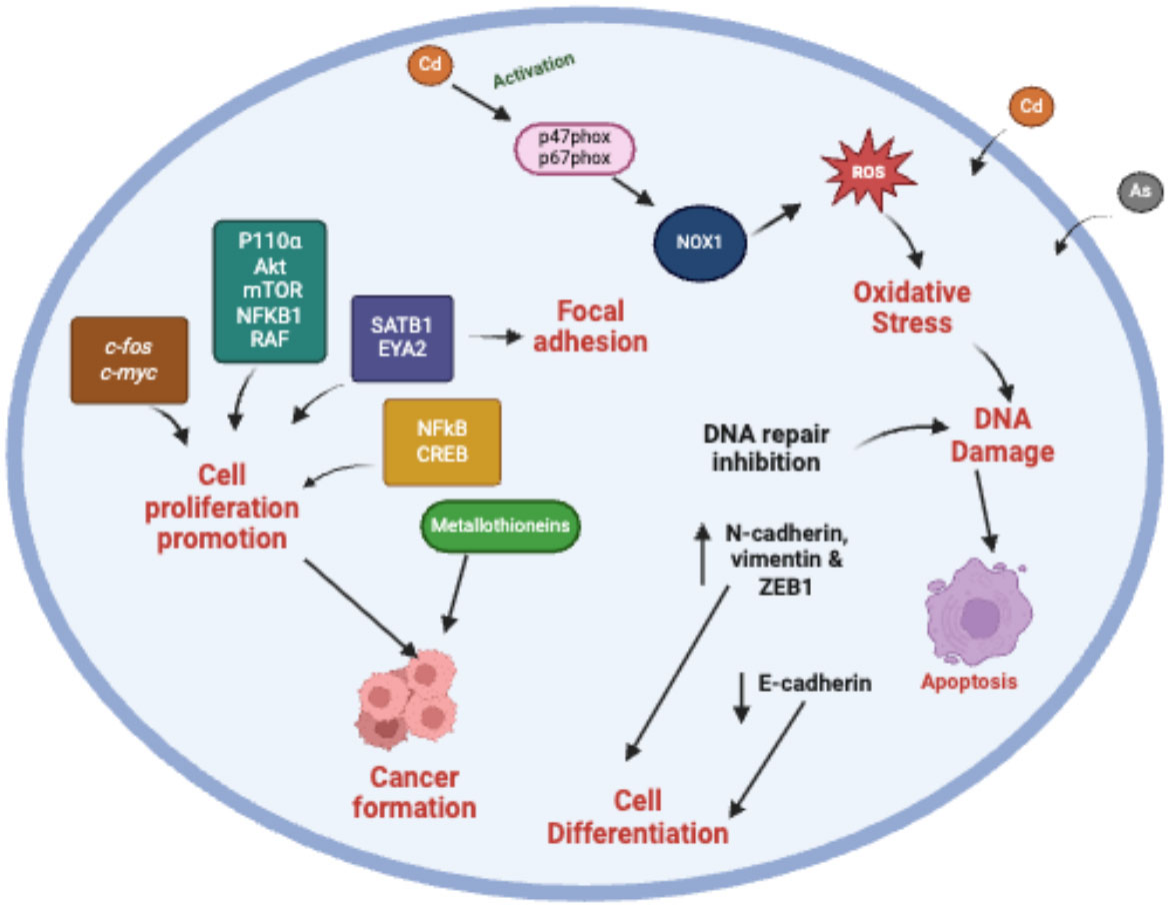
Chronic and acute metal exposure indirectly results in production of ROS which leads to oxidative stress in the cells. This activation leads to inhibition of DNA repair, DNA damage, activation of metallothioneins. Uncontrolled cell proliferation is the result of the effect of metal exposure on expression of genes such as *c-fos*, *c-myc* this activating the Ras/ERK (extracellular signal-regulated kinase) pathway. Metal exposure also results in disruption of epigenetic mechanisms such as DNA methylation by activating the PI3K/AKT/mTOR pathway result in promotion of cell proliferation.

In total, there were 22 experimental studies (**Table 3**) included in this review. The method used was mostly cell assays. The relationship of arsenic and cadmium with various cellular processes, complexes and genes that work together to result in carcinogenesis were highlighted in the studies reviewed. This includes the induction of Erk (p44/42) and Mek 1/2 which represents the Erk/MAPK pathway activation (49, 50); Modulation of the phosphoinositide 3 kinase (PI3K/Akt) pathway by oncogene induction such as P110α, Akt, mTOR, NFKB1 and RAF (51); the increased expression of KRAS thus activating the RAS/ERK pathway (52); the modulation of epithelial-mesenchymal transition (EMT) related markers, N-cadherin, vimentin, E-cadherin and ZEB1 (53); and the activation of proto-oncogenes *c-myc*, *p53 c-jun* and *c-fos* (54, 55). Through these different cellular mechanisms cadmium and arsenic were found to promote the formation, migration, proliferation and invasion of cancer cells (**Figure 3**) (31, 51, 56–59).

One of the potential mechanisms for cadmium carcinogenesis explored in this review, is that chronic and acute exposure in cells results in oxidative stress and subsequent oxidative DNA damage which has been implicated in carcinogenesis (60–62). Oxidative stress is an imbalance between an antioxidant and pro-oxidant state in cells and tissues which results in excessive production of reactive oxygen species (ROS) (63, 64). It has been hypothesized that heavy metals such as cadmium and arsenic do not increase the production of ROS directly, but rather indirectly by disruption of essential metal homeostasis. This is because transporters in human cells that are specific for toxic metal transport (analogous to copper, zinc or other essential metal transporters) have yet to be identified (65, 66). Due to its biophysiochemical similarities to essential elements such as copper, zinc, and iron (67), it has been hypothesized that cadmium transport happens by mimicking these essential metals. Heavy metals have been shown to replace essential elements at the binding sites of their respective transporter proteins (67). For example, copper, zinc, and the metal ion binding protein metallothionein, have been implicated in the transport of cadmium (65, 66, 68, 69). Thus, the heavy metals can sometimes either inhibit enzymatic pathways or act as cofactors of the pathways (67, 70–72).

Another potential mechanism of cadmium as well as arsenic induced carcinogenesis explored in this review include the attenuation of apoptosis and alterations in gene expression (54, 55, 57, 58, 73). In one study cadmium and arsenic enhanced the expression of metallothioneins in prostate cancer cells, specifically MT1 MT2 & MT3 thus resulting in significant increase in cancer cell proliferation & invasion (58). Metallothioneins are stress-inducible cysteine-rich proteins with free radical scavenging capacity (74). Metallothioneins are associated with the maintenance of the homeostasis of the essential metal ions Zn and Cu (75). There are isoforms of metallothioneins; MT-1 and MT-2 (expressed in almost all tissues), MT-3 (brain specific), and MT-4 (expressed mainly in squamous epithelium) (76). The role of epithelial-mesenchymal transition (EMT) related markers was also explored where cadmium Cd exposure resulted in up-regulated the expressions of N-cadherin, vimentin, and ZEB1, while it down-regulated expression E-cadherin (53). Epithelial-mesenchymal transition (EMT) is a biologic process in which a polarized epithelial cell undergoes multiple biochemical changes and becomes mesenchymal cell phenotype, these changes include enhanced migratory & invasive properties, and elevated resistance to apoptosis (77). There are various markers for EMT, but those highlighted in the current review fall into three categories; they are epithelial markers (E-cadherin) and mesenchymal markers (vimentin & N-cadherin) and Zinc finger E-box binding homeobox 1 (ZEB1) which plays a critical role in the epithelial-to-mesenchymal transition (EMT) process (77, 78). Cadherins calcium dependent glycoproteins that make up what is referred to as a superfamily of adhesion molecules, which include other molecules such as integrins, selectins, and immunoglobulins. They are located on cell-surface membranes and play key roles in intercellular adhesions (79, 80).

When exploring the possible therapeutic options to combat metal-induced carcinogenesis, one experimental study explored the chemoprotective effects of psoralidin on cadmium induced prostate cancer cells (81). Psoralidin is a naturally occurring compound that has been hypothesized to have chemoprotective effects (82). Pal et al. (81) found that the addition of psoralidin to cadmium induced prostate cancer cells, resulted in an inhibition of cell growth, possibly by the inhibition of placenta specific 8 (PLAC8) expression. PLAC8 is a lysosomal protein that has been implicated in organ development and tumorigenesis (83). Another study found that resveratrol reversed the migratory and invasive ability of Cd-exposed colorectal cells (53). Resveratrol is a naturally occurring compound that can be found mainly in plants such as grapes and berries and has been found to have anti-cancer properties (84).

Finally, of the 22 experimental studies included in this review, only 4 studies did analysis on cervical cancer. There were no studies found on the effects of cadmium on cervical cancer. All four experimental studies that have been included explored the therapeutic effects of arsenic trioxide on cervical cancer cells. These studies differed from all the other studies included as they explored the anti-cancer effects of arsenic trioxide rather than a potential carcinogenic effect (85–88). These studies have been included in this review as they show a contrast to all the other studies covered in the review.

## 4 Discussion

At the start of this review, a preliminary search of MEDLINE, the Cochrane Database of Systematic Reviews and JBI Evidence Synthesis was conducted and similar reviews and meta-analysis on this topic have been referenced in this article but not included in the list of articles analyzed. One systematic review of note was published during the writing of the present review and explored a similar topic of heavy metals in biological samples of patients with breast, lung, prostate and gastric cancers (89). This systematic review explored cadmium and arsenic as risks for prostate cancer. The review, however, differs from the present one in that it did not go specifically into methodologies to determine exposure. Additionally, while the review briefly referenced the relationship that exists between soil contamination by heavy metals and lung cancer incidence, it did not explore the influence of geographical location. Another review of note explored the association of cadmium exposure and prostate cancer (90). This paper was a systematic review which included a meta-analysis and was considered by the authors of this review because it met the inclusion criteria.

During the implementation of the search strategy for this review, the reviewers realized that in attempting to answer the two research questions, there were quite a few methodologies that overlapped with each other when exploring cancer carcinogenesis. It is important to note that with the complex nature of carcinogenesis, oftentimes a mixed approach is prudent. Thus, it was important that both experimental and observational studies were highlighted in this review.

The varying results shown in the observational studies included in this review show the need for ongoing work. While a majority of studies found statistically significant relationships between cadmium and/or arsenic and CPC cancers (12, 13, 28–32, 37, 41, 47, 48, 91, 92), a small number did not find a statistically significant relationship (33–36). The ecological studies, though providing the lowest level of evidence were consistent, and showed trends in increasing cancer mortality and incidence with increasing metal concentration (41–45) which would warrant further in-depth studies to confirm.

### 4.1 Studies focused on geographical location as a risk factor for CPC cancers

Only 25% of the total number of studies reviewed focused specifically on geographical location as a risk factor for CPC. It is important to note that multiple studies have been done examining the pollution of heavy metals in the environment (7, 8, 93) and the resulting exposure to humans. More work is needed to explore the relationship between cancer (incidence, prevalence and mortality) in the population and the specific geographical location of the population affected. Additionally, it is of importance to note that some of the studies presented were ecological in nature which provided the lowest level of evidence in reviews, therefore with the use of aggregated data, establishing a direct relationship is not possible at the individual level (94). However, of importance, is that some studies (29, 41, 91, 92), reported a significant difference in incidence or survival of cancer in areas of high heavy metal concentrations even after adjusting for potential covariates.

### 4.2 Gaps in experimental research

As stated in the results, only 4 experimental studies explored the heavy metal of interest in cervical cancer, The studies covered explored the anti-cancer properties of arsenic trioxide. Arsenic trioxide (AS_2_O_3_) which is also referred to as white arsenic (95), is an old drug that was reintroduced into modern medicine due to its chemotherapeutic properties and is currently used to treat leukemia that is unresponsive to first-line treatment (96, 97). In medicine, the use of arsenic trioxide is tightly regulated, and the benefits have been found to outweigh the risks in cancer treatments (98).

Arsenic trioxide differs from environmental arsenic which has been shown to have negative health effects and has been explored in the observation and experimental studies in this review (96). The main difference between arsenic trioxide and the more toxic forms of inorganic arsenic has been hypothesized to be their metabolism in the body. Arsenic compounds are metabolized by oxidative methylation reactions in which inorganic arsenic is sequentially methylated to form mono-, di-, and trimethylated products (99, 100). Sometimes, arsenic metabolism results in arsenate (pentavalent) and arsenite (trivalent) which are the most toxic forms of arsenic implicated in carcinogenesis (44, 45).

The studies explored in this review, show that there are potential chemoprotective effects of arsenic trioxide on HPV cervical cancer cells (85–88). It would be interesting to explore the effects of inorganic arsenic on cervical cancer cells in future work.

## 5 Limitations

This review acknowledges the complexity of carcinogenesis as it relates to heavy metals and therefore, the authors acknowledge that some methodologies used to determine risks may be limited. Observational studies that collect retrospective data, rely heavily on accurate & legible record keeping. As such some records may not have details on the questions developed for the research and therefore with the possibility of large amounts of data being missing due to the information not recorded, ineligible or incomplete. Additionally, it is understood that studies exploring a specific factor may be confounded by factors not considered during the completion of analysis. We have highlighted these potential confounders in the studies where necessary.

The reviewers used a systematic search strategy to retrieve several articles to answer the research questions of this review. We acknowledge that there may have been relevant studies published in other languages that were omitted from this review. Additionally, this review did not include a search of grey literature (101). This omission of the grey literature further restricted the findings presented in the review to include only information that has been reported by scientific journals which may introduce bias in the review (102). Finally, we also acknowledge that the protocol for this review was not published and note this as limitation in the study.

## 6 Conclusions

As evidenced in this review, there has been several studies conducted that explore heavy metal exposure and the risks for carcinogenesis. The methodologies used to determine these risks are quite uniform across the various studies. The results of observational studies done, point to potential and often critical relationships between cadmium or arsenic and cancer development and in some cases, aggressiveness of the cancers of interest. The results of experimental studies done, point to multiple signaling pathways that result in carcinogenesis including the promotion of ROS production, inhibition of DNA repair, regulation of metallothioneins by miRNAs and transcription factors, promotion of cancer cell proliferation & invasion though p53 protein, E-cadherin, transcription factors, increased expression of proto-oncogenes as well as disruption of processes such DNA methylation by activation of the PI3K/AKT/mTOR pathway.

On the other hand, there is a paucity of studies dealing with the potential influence of geographical location in relation to the risks of carcinogenesis. Additionally, majority of the studies explored covered prostate and colorectal cancers, however there is a paucity of studies (both observational and experimental) exploring the relationship between cadmium or arsenic and cervical cancer.

This gap in knowledge shows that more insightful research is needed to improve on the current knowledge of heavy metals and carcinogenesis. It is understood that the relationship between heavy metals and carcinogenesis is indeed a complex one that cannot be linked to only one factor. It is the hope of the authors that as the relationship between the two are continually explored from different approaches that there will be meaningful contributions to public health policies in the approach to these cancers in terms of screening, as well as the planning of community education, environmental management plans and the sensitization about the risk factors for these cancers.

## Data Availability

The original contributions presented in the study are included in the article/**Supplementary Material**. Further inquiries can be directed to the corresponding author.
