# Supplementary material for: Heavy metal carcinogenicity: a scoping review of observational & experimental evidence
Source: Front Oncol. 2025 Aug 11;15:1569816. doi: 10.3389/fonc.2025.1569816 (PMC12375472; doi:10.3389/fonc.2025.1569816)
Supplement: Supplementary file 1 [file Table1.docx]

Supplementary Material

Appendices

# Supplementary Figures and Tables

### Appendix I: Search strategy

Table 1.0: Search strategy used for articles retrieved on PubMed, and EBSCO databases

| **Database** | **Search Strategy** |
| --- | --- |
| **PubMed** | ("cadmium"[MeSH Terms] OR "cadmium" AND ("cancers" OR "cancerated" OR "canceration" OR "cancerization" OR "cancerized" OR "cancerous" OR "neoplasms"[MeSH Terms] OR "neoplasms" OR "cancer" OR "cancers") AND ("carcinogenesis"[MeSH Terms] OR "carcinogenesis" OR "carcinogeneses")  ("arsenates"[MeSH Terms] OR "arsenates" OR "arsenic acid"[Supplementary Concept] OR "arsenic acid" OR "arsenate" OR "arsenic"[MeSH Terms] OR "arsenic" OR "arsenics" OR "arsenic (s)" OR "arsenicals"[MeSH Terms] OR "arsenicals" OR "arsenical" OR "arsenism" OR "arsenous") AND ("cancer s" OR "cancerated"[All Fields] OR "canceration" OR "cancerization" OR "cancerized" OR "cancerous" OR "neoplasms"[MeSH Terms] OR "neoplasms" OR "cancer" OR "cancers") AND ("carcinogenesis"[MeSH Terms] OR "carcinogenesis" OR "carcinogeneses")  (("cadmium"[MeSH Terms] OR "cadmium") AND ("arsenates"[MeSH Terms] OR "arsenates" OR "arsenic acid"[Supplementary Concept] OR "arsenic acid" OR "arsenate" OR "arsenic"[MeSH Terms] OR "arsenic" OR "arsenics" OR "arsenic s" OR "arsenicals"[MeSH Terms] OR "arsenicals" OR "arsenical" OR "arsenism" OR "arsenous") OR ("carcinogenesis"[MeSH Terms] OR "carcinogenesis" OR "carcinogeneses") OR ("prostatic neoplasms"[MeSH Terms] OR ("prostatic" AND "neoplasms") OR "prostatic neoplasms" OR ("prostate" AND "cancer") OR "prostate cancer") OR ("colorectal neoplasms"[MeSH Terms] OR ("colorectal" AND "neoplasms") OR "colorectal neoplasms" OR ("colorectal" AND "cancer") OR "colorectal cancer") OR ("uterine cervical neoplasms"[MeSH Terms] OR ("uterine" AND "cervical" AND "neoplasms") OR "uterine cervical neoplasms" OR ("cervical" AND "cancer") OR "cervical cancer") OR ("toxic" OR "toxical" OR "toxically" OR "toxicant" OR "toxicant s" OR "toxicants" OR "toxicated" OR "toxication" OR "toxicities" OR "toxicity"[MeSH Subheading] OR "toxicity" OR "toxicity s" OR "toxics") OR ("malign" OR "malignance" OR "malignances" OR "malignant" OR "malignants" OR "malignities" OR "malignity" OR "malignization" OR "malignized" OR "maligns" OR "neoplasms"[MeSH Terms] OR "neoplasms" OR "malignancies" OR "malignancy"). |
| **EBSCO** | ("arsenates"[MeSH Terms] OR "arsenic acid"[Supplementary Concept] OR "arsenic acid" OR "arsenate" OR "arsenic"[MeSH Terms] OR "arsenic" OR "arsenics" OR "arsenic (s)" OR "arsenicals"[MeSH Terms] OR "arsenicals" OR "arsenical" OR "arsenism" OR "arsenous") AND ("cancer s" OR "canceration" OR "cancerization" OR "cancerized" OR "cancerous" OR "neoplasms"[MeSH Terms] OR "neoplasms" OR "cancer" OR "cancers") AND ("carcinogenesis"[MeSH Terms] OR "carcinogenesis" OR "carcinogeneses"). |

### Appendix II: Search Terms

Table 2.0: Comprehensive search terms utilized on PubMed and EBSCO databases

| **Population** | **Exposure/ Risk Factors** | **Outcomes** |
| --- | --- | --- |
| Main concept:  **Cervical, Prostate and Colorectal Cancer** | Main concept:  **Carcinogenesis** | Main concept :  **Cadmium and Arsenic Exposure** |
| Synonyms/ search terms :  "prostatic neoplasms" or  "prostate cancer" or  "colorectal neoplasms" or  "colorectal cancer” or  "uterine cervical neoplasms" or "cervical cancer” | Synonyms/ search terms:  “carcinogenesis” or  “carcinogeneses’ or  "malignance" or  "malignances" or  "malignant" or  "malignants" or  "malignities" or  "neoplasms" or  "malignancies" or  "malignancy" | Synonyms/ search terms :  “cadmium” or  "arsenates" or  "arsenates" or  "arsenic acid" or  "arsenic acid" or  "arsenate" or  "arsenic" or  "arsenics" or  "arsenic's" or  "arsenicals" or  "arsenicals" or  "arsenical" or  "arsenous" |
| **MeSH terms**  "prostatic neoplasms"[MeSH Terms] OR "colorectal neoplasms"[MeSH Terms] OR "uterine cervical neoplasms"[MeSH Terms] | **MeSH terms**  carcinogenesis: "carcinogenesis"[MeSH Terms] OR "carcinogeneses"[MeSH] | **MeSH terms**  "cadmium"[MeSH Terms] OR "arsenates"[MeSH Terms] OR "arsenic acid"[Supplementary Concept] OR "arsenate" OR "arsenic"[MeSH Terms] OR "arsenicals"[MeSH Terms] |
